# Vanishing of interband light absorption in a persistent spin helix state

**DOI:** 10.1038/srep02828

**Published:** 2013-10-03

**Authors:** Zhou Li, F. Marsiglio, J. P. Carbotte

**Affiliations:** 1 Department of Physics, McMaster University, Hamilton, Ontario, Canada, L8S 4M1; 2 Department of Physics, University of Alberta, Edmonton, Alberta, T6G 2E1; 3 Canadian Institute for Advanced Research, Toronto, Ontario, Canada M5G 1Z8

## Abstract

Spin-orbit coupling plays an important role in various properties of very different materials. Moreover efforts are underway to control the degree and quality of spin-orbit coupling in materials with a concomitant control of transport properties. We calculate the frequency dependent optical conductivity in systems with both Rashba and Dresselhaus spin-orbit coupling. We find that when the linear Dresselhaus spin-orbit coupling is tuned to be equal to the Rashba spin-orbit coupling, the interband optical conductivity disappears. This is taken to be the signature of the recovery of SU(2) symmetry. The presence of the cubic Dresselhaus spin-orbit coupling modifies the dispersion relation of the charge carriers and the velocity operator. Thus the conductivity is modified, but the interband contribution remains suppressed at most but not all photon energies for a cubic coupling of reasonable magnitude. Hence, such a measurement can serve as a diagnostic probe of engineered spin-orbit coupling.

Spin-orbit coupling in semiconductors^1^ and at the surface of three dimensional topological insulators^2^,^3^,^4^,^5^,^6^,^7^,^8^ where protected metallic surface states exist, plays a crucial role in their fundamental physical properties. Similarly pseudospin leads to novel properties in graphene^9^,^10^,^11^ and other two dimensional membranes, such as single layer *MoS*_2_^12^,^13^,^14^,^15^,^16^,^17^ and silicene^18^,^19^,^20^,^21^,^22^. In particular *MoS*_2_ has been discussed within the context of valleytronics where the valley degree of freedom can be manipulated with the aim of encoding information in analogy to spintronics. Spin-orbit coupling has also been realized in zincblende semiconductor quantum wells^23^,^24^,^25^ and neutral atomic Bose-Einstein condensates^26^ at very low temperature^27^.

In some systems both Rashba^28^ and Dresselhaus^29^ spin-orbit coupling are manipulated, the former arising from an inversion asymmetry of the grown layer while the latter comes from the bulk crystal. In general spin-orbit coupling will lead to rotation of the spin of charge carriers as they change their momentum, because SU(2) symmetry is broken. In momentum space this has been observed by angle-resolved photoemission spectroscopy (ARPES) as the phenomenon of spin momentum locking. In a special situation when the strength of Rashba and Dresselhaus spin-orbit coupling are tuned to be equal, SU(2) symmetry is recovered and a persistent spin helix state is found^23^,^24^,^25^. This state is robust against any spin-independent scattering. However it will be potentially destroyed by the cubic Dresselhaus term which is usually tuned to be negligible.

To describe these effects we consider a model Hamiltonian describing a free electron gas with kinetic energy given simply by ℏ^2^*k*^2^/(2*m*), which describes charge carriers with effective mass *m*. We also include spin-orbit coupling terms, with linear Rashba (*α*_1_) and Dresselhaus (*β*_1_) couplings, along with a cubic Dresselhaus (*β*_3_) term. The Hamiltonian is 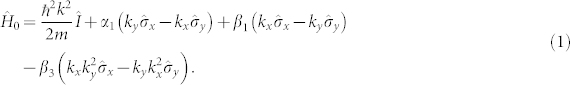
Here 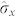, 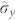 and 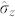 are the Pauli matrices for spin (or pseudospin in a neutral atomic Bose-Einstein condensate) and 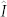 is the unit matrix. For units we use a typical wave vector *k*_0_ ≡ *mα*_0_/*ℏ*^2^ with corresponding energy 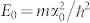, where *α*_0_ is a representative spin-orbit coupling which has quite different values for semiconductors (*α*_0_/*ℏ* ≈ 10^5^ *m*/*s*, estimated from Ref. 25) and cold atoms (*α*_0_/*ℏ* ≈ 0.1 *m*/*s*, estimated from Ref. 26). The mass of a cold atom is at least 1000 times heavier than that of an electron and the wavelength of the laser used to trap the atoms is at least 1000 times (estimated from Ref. 26) larger than the lattice spacing in semiconductors.

In this report we study the dynamic longitudinal optical conductivity of such a spin-orbit coupled 2D electron gas. We find that the interband optical absorption will disappear when the Rashba coupling is tuned to be equal to the Dresselhaus coupling strength. We discuss the effect of nonlinear (cubic) Dresselhaus coupling on the shape of the interband conductivity and the effect of the asymmetry between the conduction and valence band which results from a mass term in the dispersion curves.

## Results

**Figure 1:**
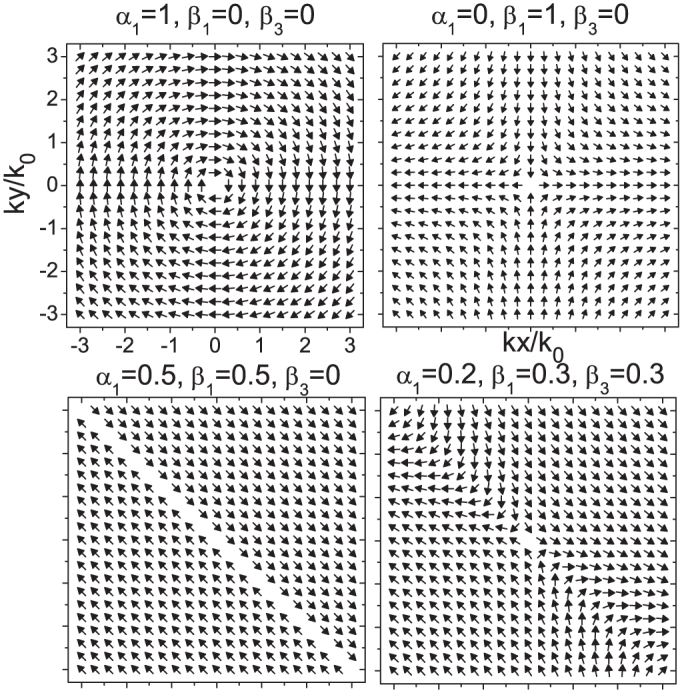
Spin texture in the conduction band as a function of momentum *k_x_*/*k*_0_, *k_y_*/*k*_0_ for various values of Rashba (*α*_1_), Dresselhaus (*β*_1_), and cubic Dresselhaus (*β*_3_) spin-orbit coupling. In the case of purely Rashba coupling (upper left frame), the spin is locked in the direction perpendicular to momentum, while for linear Dresselhaus coupling (upper right frame) the y-component of spin is of opposite sign to that of its momentum. For the persistent spin helix state (lower left frame) all spins are locked in the 3*π*/4 direction and oppositely directed on either side of this critical direction. The lower right frame shows the spin texture for a case with all three kinds of coupling.

We compute the optical conductivity (see Methods section) as a function of frequency, for various electron fillings and spin-orbit coupling strengths. In all our figures we will use a dimensionless definition of spin-orbit coupling; for example, the choice of values designated in the lower right frame of Fig. 1, *α*_1_ = 0.2, *β*_1_ = 0.3, and *β*_3_ = 0.3, really means *α*_1_/*α*_0_ = 0.2, *β*_1_/*α*_0_ = 0.3, and 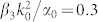.

**Figure 2:**
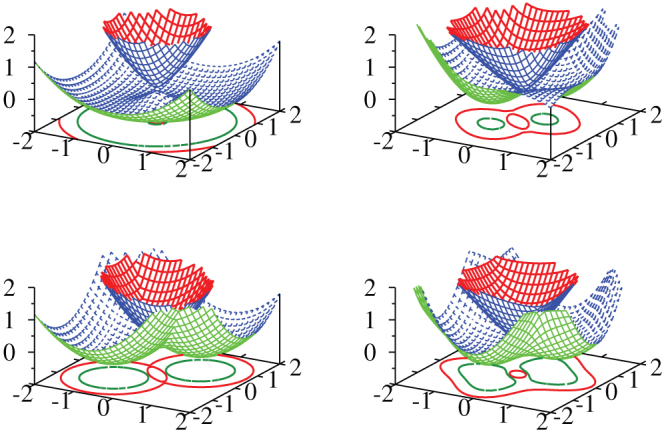
Band structure of the conduction and valence band (Eq. (10)) as a function of momentum *k_x_*/*k*_0_, *k_y_*/*k*_0_ for various values of Rashba (*α*_1_), Dresselhaus (*β*_1_), and cubic Dresselhaus (*β*_3_) spin-orbit coupling. The left two panels are for pure Rashba *α*_1_ = 1.0, *β*_1_ = 0.0, *β*_3_ = 0.0 (top panel) and Rashba equals to Dresselhaus *α*_1_ = 0.5, *β*_1_ = 0.5, *β*_3_ = 0.0 (bottom panel). The right two panels are for *α*_1_ = 0.4, *β*_1_ = 0.4, *β*_3_ = 0.3 (top panel) and *α*_1_ = 0.2, *β*_1_ = 0.8, *β*_3_ = 0.3 (bottom panel). The dispersion curves are profoundly changed from the familiar Dirac cone of the pure Rashba case when *β*_1_ and *β*_3_ are switched on. In the contour plots, red refers to energy 0.2*E*_0_ and dark green refers to energy −0.2*E*_0_.

In Fig. 1 we plot the spin direction in the conduction band as a function of momentum for several cases. The top left frame is for pure Rashba coupling, in which case spin is locked to be perpendicular to momentum^2^ as has been verified in spin angle-resolved photoemission spectroscopy studies^30^,^31^,^32^,^33^. The top right frame gives results for pure linear Dresselhaus coupling (no cubic term *β*_3_ = 0). The spin pattern is now quite different; the direction of the spin follows the mirror image of the momentum about the x-axis. The lower left frame for equal linear Rashba and Dresselhaus coupling is the most interesting to us here. All spins are locked in one direction, namely *θ* = 3*π*/4 with those in the bottom (upper) triangle pointing parallel (anti-parallel) to the 3*π*/4 direction, respectively. This spin arrangement corresponds to the persistent spin helix state of Ref. 23,24,25. The condition *α*_1_ = *β*_1_ and *β*_3_ = 0 is a state of zero Berry phase^34^ and was also characterized by Li *et al.*^35^ as a state in which the spin transverse “force” due to spin-orbit coupling cancels exactly. Finally the right lower frame includes a contribution from the cubic Dresselhaus term of Eq. (1) and shows a more complex spin arrangement. Spin textures have been the subject of many recent studies^30^,^31^,^32^,^33^,^36^. In Fig. 2 we present results for the dispersion curves in the conduction and valence band *E*_+/–_(*k*) of Eq. (10) as a function of momentum *k*. The two left panels are pure Rashba (top) and Rashba equals to Dresselhaus (bottom, see also Fig. 1 of Ref. 37 where only the contour plots of the valence band is shown). The two right panels include the Dresselhaus warping cubic term which profoundly affects the band structure.

The optical conductivity is obtained through transitions from one electronic state to another. In general these can be divided into two categories — transitions involving states within the same band, and interband transitions. Here we focus on interband transitions; the interband optical conductivity is given by 
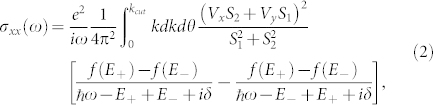
where 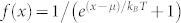 is the Fermi-Dirac distribution function with *μ* the chemical potential. For *β*_3_ = 0 and *β*_1_ = *α*_1_, we have a cancellation in the optical matrix element, *V_x_S*_2_ + *V_y_S*_1_ = 0; remarkably the interband contribution vanishes. This result is central to our work and shows that in the persistent spin helix state the interband contribution to the dynamic longitudinal optical conductivity vanishes. This is the optical signature of the existence of the spin helix state which exhibits remarkable properties. With *β*_3_ = 0 the optical matrix element is 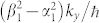. Thus, pure Rashba or pure (linear) Dresselhaus coupling will both lead to exactly the same conductivity although the states (and spin texture) involved differ by a phase factor of *π*. When they are both present in equal amounts this phase leads to a cancelation which reduces the interband transitions to zero as the two contributions need to be added before the square is taken. Of course the joint density of states, widely used to discuss optical absorption processes, remains finite. It is given by 
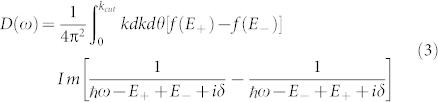
and will be contrasted with the interband optical conductivity below.

**Figure 3:**
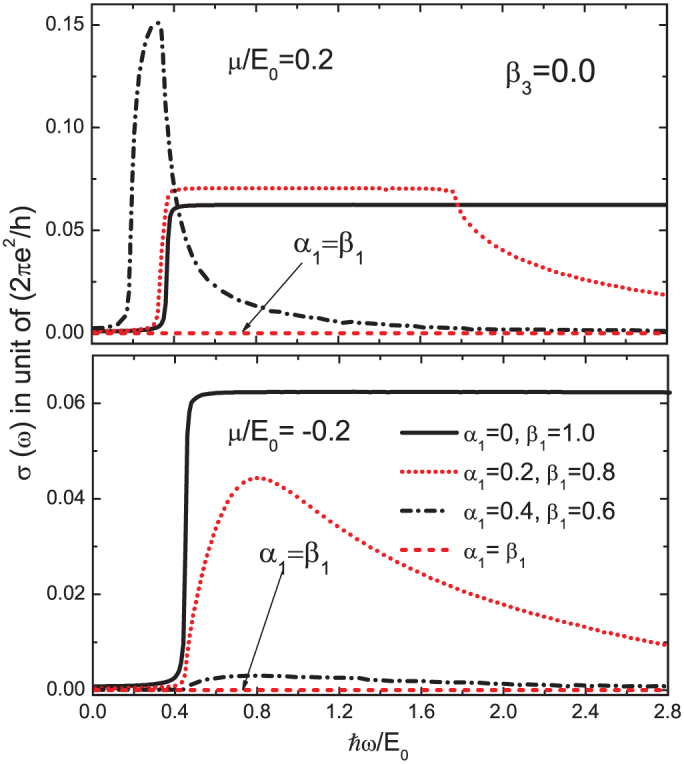
The interband contribution to the longitudinal optical conductivity of Eq. (2) for various values of *α*_1_ and *β*_1_ as labeled, with *β*_3_ set to zero. In the top frame the chemical potential was set at *μ*/*E*_0_ = 0.2 and in the bottom *μ*/*E*_0_ = −0.2.

We first focus on the case *β*_3_ = 0. The interband conductivity is shown in Fig. 3 as a function of frequency for positive (top frame) and negative (bottom frame) chemical potential (*μ*/*E*_0_ = ± 0.2). It is clear that there is a considerable difference between the two cases, and there is also considerable variation with the degree of Rashba vs. Dresselhaus coupling. This will be discussed further below. Most important is that for equal amounts of Rashba and Dresselhaus coupling, the interband conductivity is identically zero for all frequencies.

**Figure 4:**
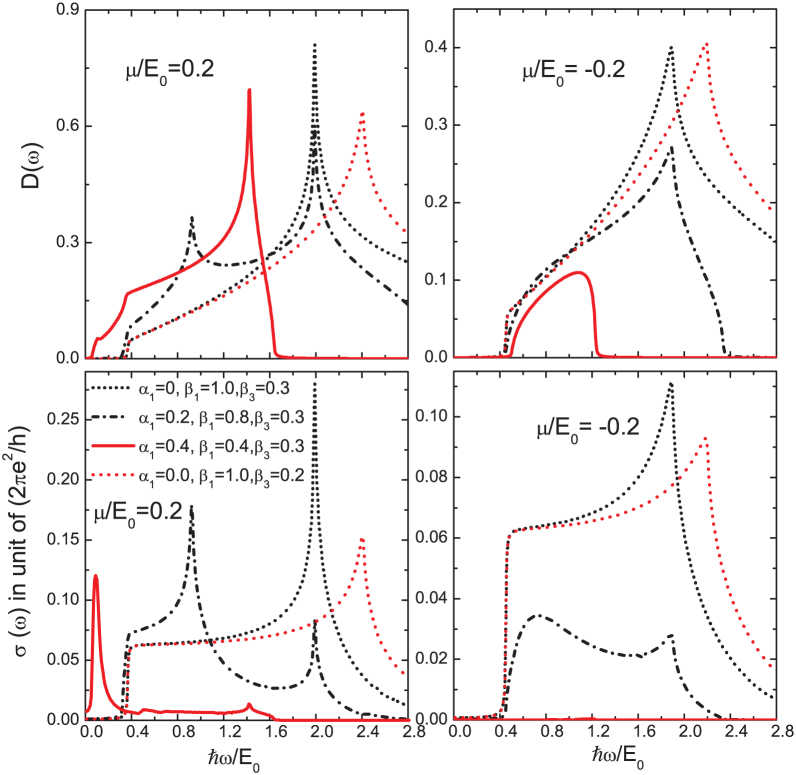
Joint density of states *D*(*ω*) (top two panels) defined in Eq. (3) which involves the same transitions as does the interband conductivity (bottom two panels) of Eq. (2) but without the critical weighting 
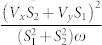
. Left column is for positive chemical potential *μ*/*E*_0_ = 0.2 and the right for −0.2.

What is the impact of a finite value of *β*_3_? In Fig. 4 we show both the joint density of states (top two panels) and the interband conductivity (bottom two panels) for non-zero *β*_3_ for *μ*/*E*_0_ = 0.2 (left panels) and *μ*/*E*_0_ = –0.2 (right panels). Various combinations of *α*_1_, *β*_1_ and *β*_3_ are shown as labeled on the figure. There is a striking asymmetry between positive and negative values of the chemical potential. This asymmetry has its origin in the quadratic term ℏ^2^*k*^2^/(2*m*) of the Hamiltonian (1) which adds positively to the energy in both valence and conduction band while the Dirac like contribution is negative (*s* = –1) and positive (*s* = +1) respectively [see Eq. (10)]. While the quadratic piece drops out of the energy denominator in Eq. (2) it remains in the Fermi factors *f*(*E*_+_) and *f*(*E*_–_).

**Figure 5:**
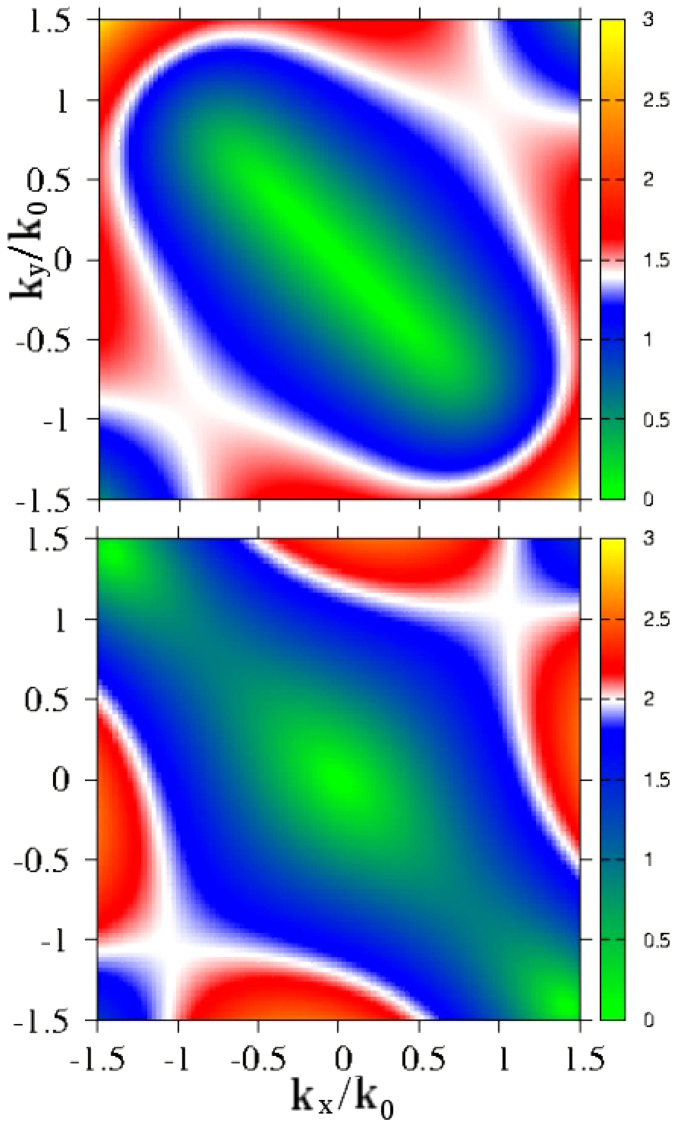
Color contour plot of the energy difference 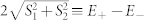, as a function of momentum (*k_x_*, *k_y_*) in units of *k*_0_ for *α*_1_ = 0.4, *β*_1_ = 0.4, *β*_3_ = 0.3 (top panel) and *α*_1_ = 0.2, *β*_1_ = 0.8, *β*_3_ = 0.3 (bottom panel).

Several features of these curves are noteworthy. They all have van Hove singularities which can be traced to extrema in the energy difference 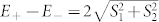. Taking *β*_3_ = 0 for simplicity, this energy becomes 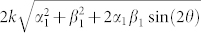 which depends on the direction (*θ*) of momentum **k**, but has no minimum or maximum as a function of |**k**| = *k*. To get an extremum one needs to have a non-zero cubic Dresselhaus term. This gives dispersion curves which flatten out with increasing values of *k*. The dependence of the energy *E*_+_ – *E*_–_ on momentum is illustrated in Fig. 5 where we provide a color plot for this energy as a function of *k_x_*/*k*_0_ and *k_y_*/*k*_0_ for two sets of spin-orbit parameters *α*_1_ = 0.4, *β*_1_ = 0.4, *β*_3_ = 0.3 (top panel) and *α*_1_ = 0.2, *β*_1_ = 0.8, *β*_3_ = 0.3 (bottom panel). Note the saddle points correspond to the most prominent van Hove singularities in the joint density of states (and conductivity) in Fig. 4. The van Hove singularities are at about 1.4*E*_0_ (*k_x_* = *k_y_* in the momentum space) in the top frame of Fig. 5 and at about 2*E*_0_ (*k_x_* = *k_y_*) and 0.9*E*_0_ (*k_x_* = –*k_y_*) in the bottom.

## Discussion

The optical conductivity is often characterized by the joint density of states, *D*(*ω*), which has a finite onset at small energies. This is well known in the graphene literature where interband transitions start exactly at a photon energy equal to twice the chemical potential. Here this still holds approximately in all the cases considered in Fig. 4 except for the solid red curve in the two left side frames. In this case *α*_1_ = *β*_1_ = 0.4 and *β*_3_ is non zero. If *β*_3_ is small the energy 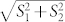 would be approximately equal to 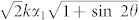, which is zero for *θ* = 3*π*/4, the critical angle in the spin texture of the lower left frame of Fig. 1 for which all spins are locked in this direction. This means that only the quadratic term *ℏ*^2^*k*^2^/(2*m*) and cubic Dresselhaus term contribute to the dispersion curve in this direction and there is no linear (in *k*) graphene-like contribution. Thus, the onset of the interband optical transition no longer corresponds to *ω* = 2*μ*.

Considering the case of positive *μ*, for the direction *θ* = 3*π*/4, (*k*/*k*_0_)^2^/2 + *β*_3_(*k*/*k*_0_)^3^ is the dominant contribution to the energy which is equal to *μ*/*E*_0_ and the minimum photon energy is now 2*β*_3_(*k*/*k*_0_)^3^, which could be very small as is clear from the figure. For negative values of *μ* the onset is closer to 2|*μ*|/*E*_0_ because in this case the momentum at which the chemical potential crosses the band dispersion is given by (*k*/*k*_0_)^2^/2 − *α*_1_(*k*/*k*_0_) = −*μ*/*E*_0_ (the cubic term is ignored because it is subdominant for small *k*/*k*_0_ compared to the linear term). Now the photon energy onset will fall above 2|*μ*|/*E*_0_, at a value dependent on *α*_1_.

While the optical conductivity Eq. (2) requires a non-zero joint density of states Eq. (3), the additional weighting of (*V_x_S*_2_ + *V_y_S*_1_)^2^ in *σ_xx_*(*ω*) can introduce considerable changes to its *ω* dependence^38^ as we see in Fig. 3 and Fig. 4. In the top frame of Fig. 3, *β*_3_ = 0 and there are no van Hove singularities because the Dirac contribution to the dispersion curves simply increases with increasing *k*. The solid black and dashed red curves both reduce to the pure graphene case with onset exactly at 2*μ* and flat background beyond. The dotted red curve for mixed linear Dresselhaus and Rashba is only slightly different. The onset is near but below 2*μ* and the background has increased in amplitude. It is also no longer completely flat to high frequency; instead it has a kink near *ℏω*/*E*_0_ ≈ 1.7 after which it drops. The dash-dotted black curve for *α*_1_ = 0.4 and *β*_1_ = 0.6 has changed completely with background reduced to near zero but with a large peak corresponding to an onset which has shifted to a value much less than 2*μ*. Finally for *α*_1_ = *β*_1_ the entire interband transition region is completely depleted as we know from Eq. (2).

In Fig. 4 there is (non-zero) cubic Dresselhaus coupling present. The solid red curves, for which *α*_1_ = *β*_1_ but with *β*_3_ = 0.3 illustrate that the conductivity on the left (positive *μ*) is non-zero, and *β*_3_ = 0 is necessary for a vanishing interband conductivity at all photon energies. We see, however, that these transitions have been greatly reduced below what they would be in graphene for all photon energies except for a narrow absorption peak at *ω* much less than 2*μ*. For negative values of *μ*, on the other hand, even with *β*_3_ ≠ 0 the conductivity is zero.

The experimental observation of such a narrow low energy peak together with high energy van Hove singularities could be taken as a measure of nonzero *β*_3_. It is interesting to compare these curves for the conductivity with the joint density of states (lower frames). The color and line types are the same for both panels. The onset energy as well as energies of the van Hove singularities are unchanged in going from the joint density of states to the conductivity. Also, as is particularly evident in the dotted black and short dashed red curves the 1/*ω* factor in *σ_xx_*(*ω*) leads to a nearly flat background for the conductivity as compared with a region of nearly linear rise in the density of states. This is true for both positive and negative values of *μ*.

In conclusion we have calculated the interband longitudinal conductivity as a function of photon energy for the case of combined Rashba and Dresselhaus spin-orbit coupling. We have also considered the possibility of a cubic Dresselhaus contribution. We find that in the persistent spin helix state when the spins are locked at an angle of 3*π*/4 independent of momentum, which arises when the linear Rashba coupling is equal to the linear Dresselhaus coupling, the interband optical transitions vanish and there is no finite energy absorption from these processes. Only the Drude intraband transitions will remain. When the cubic Dresselhaus term is nonzero the cancelation is no longer exact but we expect interband absorption to remain strongly depressed for photon energies above 2*μ* as compared, for example, to the universal background value found in single layer graphene. We propose interband optics as a sensitive probe of the relative size of Rashba and Dresselhaus spin orbit coupling as well as cubic corrections.

## Methods

The optical conductivity is given by 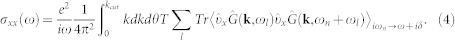
Here *T* is the temperature and *Tr* is a trace over the 2 × 2 matrix, and *ω_n_* = (2*n* + 1)*πT* and *ω_l_* = 2*lπT* are the Fermion and Boson Matsubara frequencies respectively with *n* and *l* integers. To get the conductivity which is a real frequency quantity, we needed to make an analytic continuation from imaginary *iω_n_* to *ω* + *iδ*, where *ω* is real and *δ* is an infinitesimal. The velocity operators 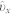 and 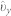 are given by 
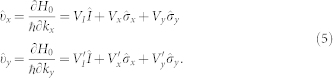
Here *V_I_* = *ℏk_x_*/*m*, 
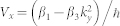
, *V_y_* = (−*α*_1_ + 2*β*_3_*k_y_k_x_*)/*ℏ*, 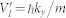, 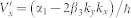 and 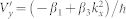.

The Green's function can be written as^39^

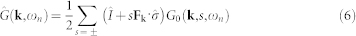
where 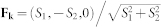, 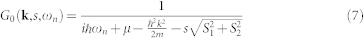
and 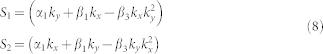
The wave function is given by 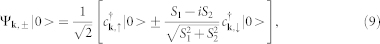
with corresponding eigenvalues 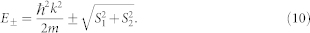
Here 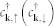 creates a particle with momentum **k** and spin up (down). The spin expectation values work out to be 
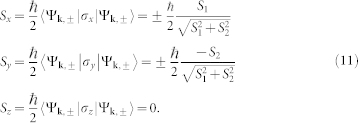
These formulas allow us to calculate the spin texture, as well as the optical conductivity as given in Eq. (2).

## Author Contributions

Z.L. carried out the calculations, and all authors, Z.L., F.M. and J.P.C. contributed equally to the development of the work.
